# Diagnosis of fast-growing thoracic aneurysm with microscopic evidence of dissection over 6 months follow-up in an asymptomatic middle aged gentleman: a case report

**DOI:** 10.1186/s12872-022-02687-6

**Published:** 2022-06-06

**Authors:** Mohammadbagher Sharifkazemi, Mohammadhassan Nemati, Seyed Mohammad Owji, Leila Ahmadi

**Affiliations:** 1 Department of Cardiology, Nemazee Hospital, Shiraz University of Medical Sciences, Nemazee Square, Shiraz, 71936-13311 Iran; 2 Department of Cardiac Surgery, Medical School, Shiraz University of Medical Sciences, Shiraz, Iran; 3 Department of Pathology, School of Medicine, Shiraz University of Medical Sciences, Shiraz, Iran

**Keywords:** Isolated non-compaction of the ventricular myocardium, Aortic aneurysm, Non-compaction of left ventricular myocardium with congenital heart defects, Fast-growing aortic aneurysm, Subclinical dissecting aneurysm

## Abstract

**Background:**

Thoracic aortic aneurysm (TAA), is a pathological dilatation of the aortic segment with the tendency to expand, dissect or rupture, and risk of mortality. The progression rate is mainly slow. As the risk of rupture increases with the size of the aortic diameter, it is important to diagnose TAA appropriately to prevent mortality.

**Case presentation:**

Here, we present a case with a fast-growing TAA, complicated by subclinical dissection in a middle-aged gentleman, associated with non-compaction left ventricle, diagnosed 6 months after the first diagnosis of this co-occurrence, successfully managed by an uneventful surgical procedure. The pathological examination was the key to the diagnosis of this concealed phenomenon, i.e. a fast-growing aortic aneurysm complicated by subclinical dissection.

**Conclusion:**

This case report emphasizes the importance of close follow-up of patients with fast-growing TAA for considering remote possibility of this silent life-threatening disease; subclinical dissecting aneurysm, especially in patients with other cardiac comorbidities. Although imaging modalities can help accurate diagnosis, in cases with fast-growing TAA, we should not wait for imaging signs of dissection and/or rupture.

## Background

Thoracic aortic aneurysm (TAA), the arterial diameter of 1.5-fold the maximum value, is a pathological dilatation of the aortic segment and degenerative process involving all layers of the vessel wall with a tendency to gradually expand and rupture [1]. It has a prevalence of 5–10 per 100,000 person-years [2]. TAA is mainly a slow-growing disease with an estimated growth rate of 0.12 cm/y, varying based on the aneurysm location, slower in ascending than descending aorta [3].

As the risk of rupture increases with the size of the aortic diameter, it is important to diagnose TAA appropriately to prevent mortality [4]. Although the imaging modalities [such as transthoracic echocardiography (TTE), transesophageal echocardiography (TEE), computer tomographic angiography scan (CTA), and cardiac magnetic resonance (CMR) imaging] have helped accurate diagnosis in recent years, the silent and asymptomatic presence/progression of TAA makes diagnosis challenging [5]. Moreover, co-occurrence of of this lethal condition, TAA, with other cardiac diseases may worsen the situation.

Here, we present a patient with missed diagnosis of left ventricular non-compaction (LVNC) until the 6^th^ decade of his life with a fast-growing TAA accompanied by subclinical aortic dissection, diagnosed by microscopic examination of the surgically excised aorta, during 6-month follow-up, while the pre-operative clinical symptoms and signs as well as TEE examination were not in favor of aortic dissection.

## Case report

A 56-year-old gentleman, was referred to Cardiology Outpatient Clinic at Shiraz Central Hospital with dyspnea on exertion since 3 months earlier, which became worse over the time. His parents had no consanguineous marriage; moreover, he had neither history of underlying disease nor major modifiable cardiovascular risk factors in him as well as his first relatives. On the physical examination, signs of left sided herat failure was detected.

**Fig. 1:**
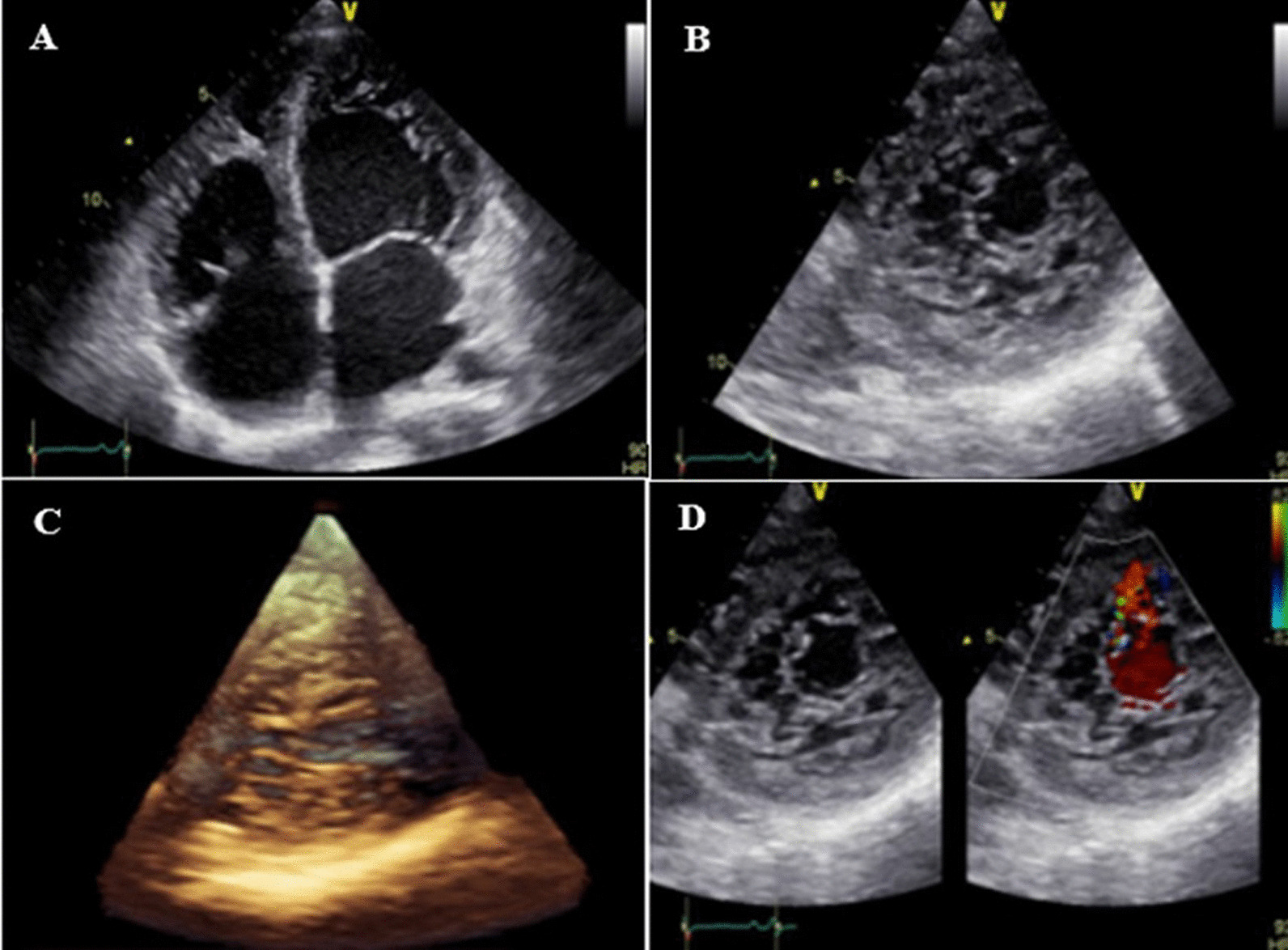
**A**–**D** Two- and three-dimensional transthoracic echocardiographic findings; apical four chamber view (**A**), apical SAX view (**B**, **C**), illustrating hypertrabeculated apical portions, in addition to deep intertrabecular recesses. Evidence of the direct blood flow from the ventricular cavity into deep intertrabecular recesses via color Doppler echocardiography (**D**)

**Fig. 2:**
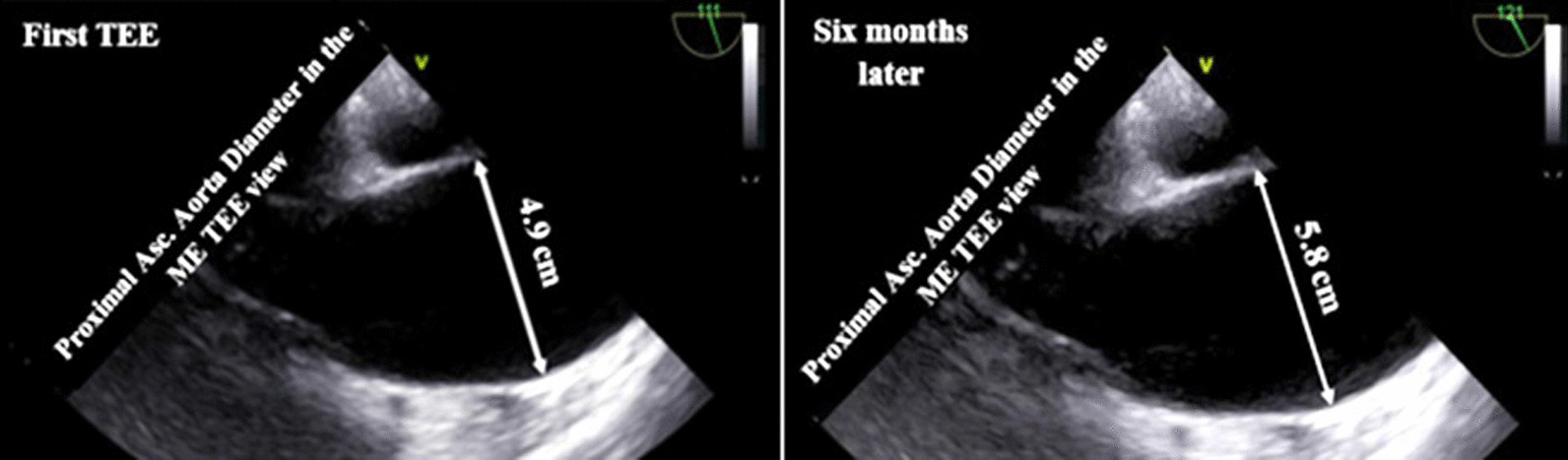
Transesophageal echocardiographic findings of baseline (left) and 6 months later (right); follow-up test showed fast-growing aortic root diameter that reached from 4.9 to 5.8 cm

On electrocardiogram, he had sinus tachycardia, low voltage QRS complex, and poor R progression in the precordial leads. TTE (two- and three-dimensional) showed left ventricular non-compaction in the presence of severe global hypokinesia. The left ventricular ejection fraction (LVEF), calculated by Simpson’s method, was 16%, myocardial non-compaction/compaction ratio (NC/C) = 2.2, a diameter of compacted myocardium in the apicolateral and apical segments was 10 mm, in addition to the presence of a PFO. All valves, including the aortic valve, were competent (Fig. 1A–D). Dilated proximal ascending aorta was up to 4.9 cm, and effacement was noted as well, confirmed by TEE (Fig. 2). The patient refused to perform CMR, because of claustrophobia. The results of whole-exome sequencing (WES) showed three heterozygous mutations in the *DSP* (e.3857_3859del:p.1286_1287del.), *TTN* (c.C80492T:p.P26831L), and *DSC2* (c.A1886G:p.N629S). These identified mutations were not reported so far and classified as a variants of uncertain significance (VUS).

**Fig. 3:**
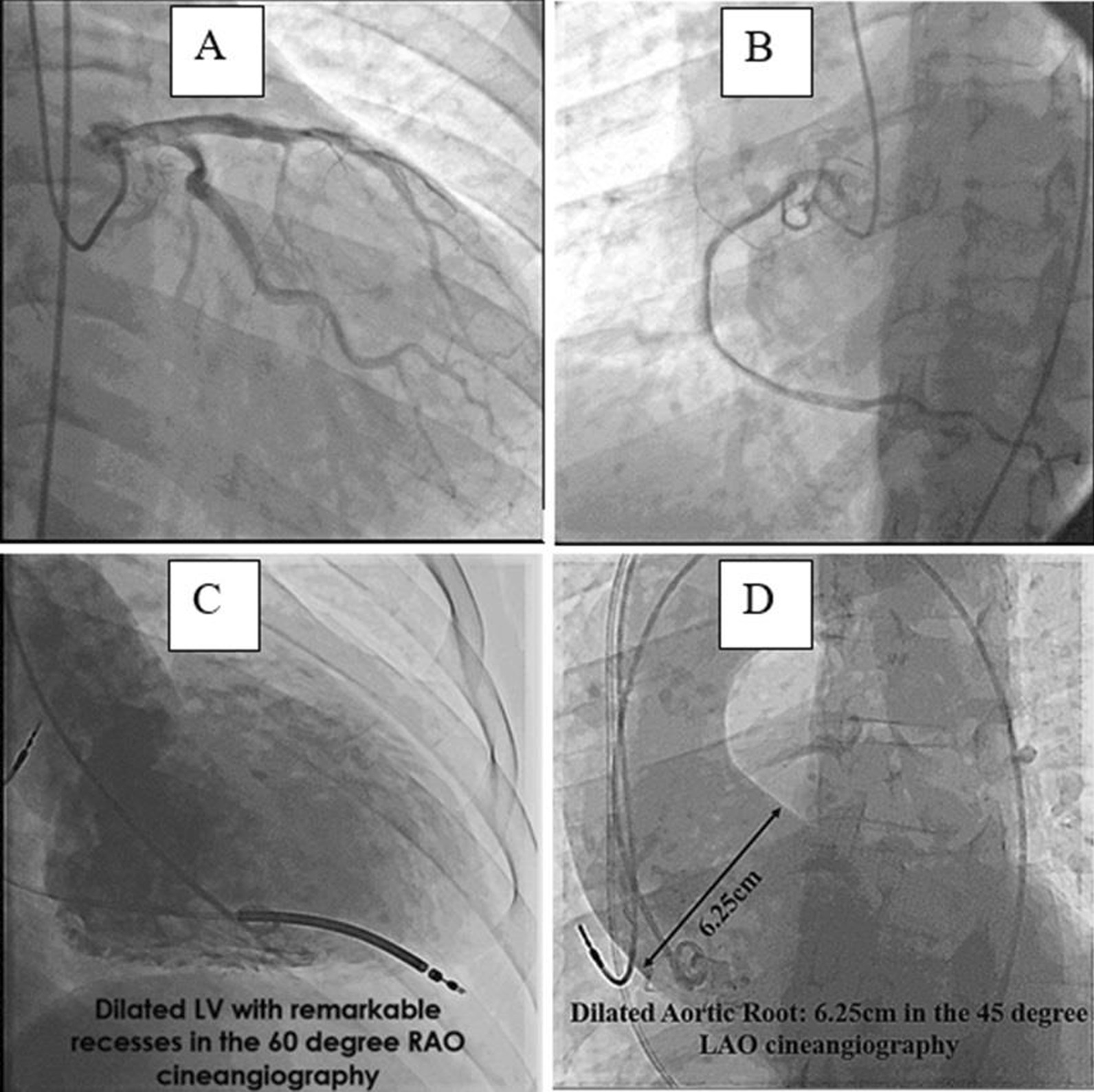
**A**–**D** Coronary angiography illustrating normal left and right coronaries (**A**, **B**). Left ventricular cineangiography showing dilated left ventricle with remarkable hypertrabeculation and deep recesses. The ICD leads are seen in the exact place (**C**). Aortic root cineangiography showing markedly dilated aortic root (**D**)

The patient received carvedilol 6.25 mg twice a day, spironolactone 25 mg once a day, furosemide 60 mg once daily, warfarin 5 and 2.5 mg every other day. At first and in the first month, because he refused to be hospitalized, the patient had outpatient follow-up once per week, and then once per month. After 3 weeks and partial recovery, the patient underwent computed tomographic coronary angiography (CCTA), which showed patent epicardial coronary arteries. Then, implantation of a cardioverter defibrillator (ICD) was performed. Six-month follow-up TTE as well as TEE showed a fast growing aortic root aneurysm that reached to 5.8 cm, although the patient was receiving beta-blockers and had no clinical symptoms. The baseline and follow-up TEE results are compared in Fig. 2. The patient was scheduled for cardiac surgery, and according to the request of the cardiac surgeon, we did coronary angiography, LV, and aortic root cineangiography, which the results showed patent epicardial coronaries, dilated LV with remarkable recesses, dilated aortic root (6.25 cm), and competent tricuspid aortic valve (Fig. 3). Pre-operative TEE showed no evidence of a dissecting aneurysm.

So, the patient underwent valve-sparing aortic root replacement surgery (David procedure), and the ascending aortic aneurysm was seen with a diameter of about 6.5 cm. After resection of the ascending aorta, valve-sparing aortic root replacement with gel cell Dacron graft number 28 was done. Finally, PFO was closed directly, and cardiopulmonary bypass was weaned off with inotrope. The perioperative and postoperative trans-esophageal echocardiography showed normal functioning native aortic valve with neither aortic insufficiency nor stenosis.

**Fig. 4:**
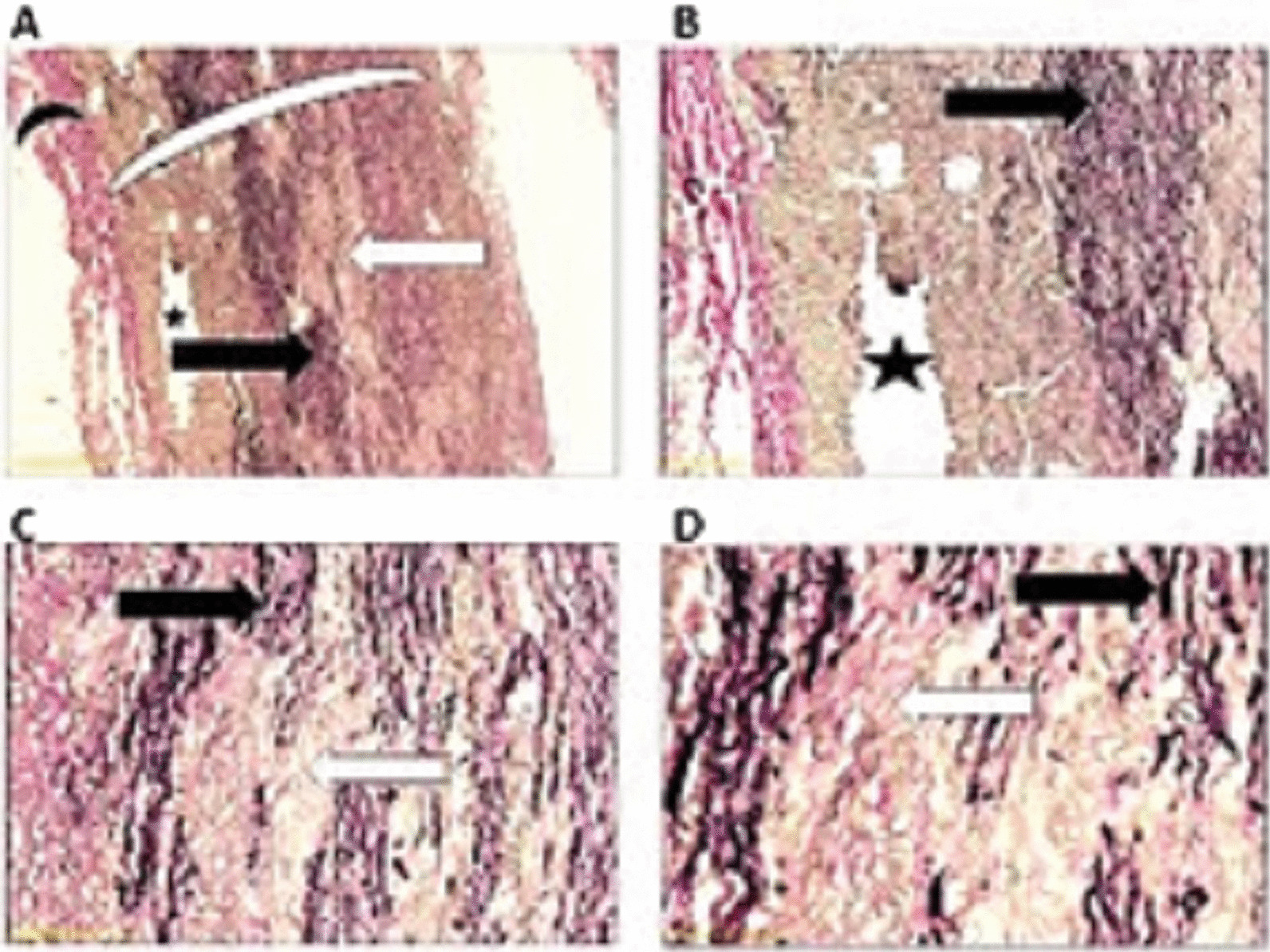
Microscopic findings of reticulum stained aortic wall of aneurysmal part of the excised aortic root, demonstrating a luminal formation within tunica media (black asterisk) with patchy fragmentation, splitting, and destruction of reticulum fibers in the tunica media (black arrow). There is an extensive patchy pale area in the tunica media which show the absence of the reticulum tissue (white arrow) (Reticulum stain, A*40, B*100, C*200, D*400)

The gross appearance of the excised aortic aneurysm specimen showed uniform fusiform dilation of the aortic wall. Light microscopic findings of reticulum stained aortic wall of aneurysmal part of the excised aortic root, demonstrating a luminal formation within tunica media with patchy fragmentation, splitting, and destruction of reticulum fibers in the tunica media, without fibrosis or inflammatory cells that approved dissecting aortic aneurysm (Fig. 4).

**Fig. 5:**
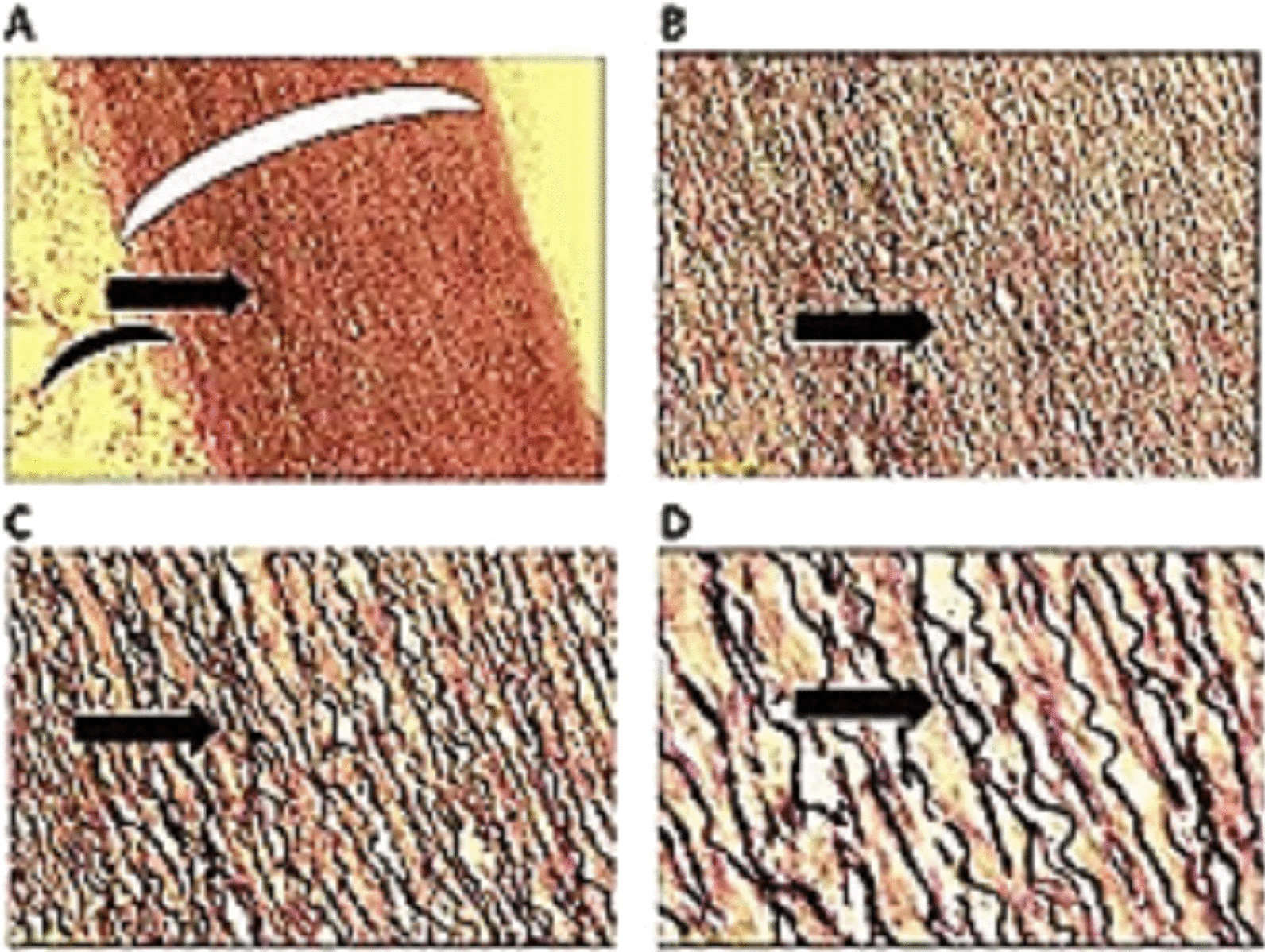
Microscopic findings of the normal part of the excised aortic root, showing normal elastic tissue in the tunica media; the reticulum fibers are normal, located very close to each other and in a regular paracell wavy pattern (black arrow), with intervening smooth muscle cells. The tunica media thickness is normal and uniform (large white eyebrow). Black small eyebrow shows normal intima. (Reticulum stain A*40, B*100, C*200, D*400)

Microscopic appearance of normal aortic wall is showing for comparison (Fig. 5).

The patient had an uneventful postoperative course and was discharged 4 days after the surgery. During his admission, he appeared to be a quite pleasant and cooperative patient who established a friendly patient-physician relationship. Post-operative follow-up arranged as 1 month later and then every 3 months over the following year. At the follow-up visits, the patient had no clinical symptoms related to heart failure. Also, the result of follow-up transthoracic echocardiographies were acceptable. The previous medications were prescribed, and the dose of furosemide was changed to 40 mg daily.

## Discussion and conclusion

We presented a patient who who presented with TAA, and LVNC in whom a fast-growing TAA was diagnosed 6 months after the initial presentation and successfully managed by David’s procedure. Although we did not observe the signs of dissection, including intimal and/or intimomedial flap to indicate urgent surgery [6], an urgent surgical replacement was performed for the patient because of the high risk of rupture [7]. The pathological examination was the key to enforce accurate and on-time diagnosis of subclinical dissecting aneurysms.

As most cases of TAA are indolent and have a slow growth rate, it is important that cardiologists and surgeons pay greater attention to this phenomenon, fast-growing TAA, by keeping in mind the possibility of the presence of a microscopic dissecting aneurysm. A various mean growth rate of TAA is reported, such as 1.19 and 0.59 mm/y [8], and 0.96 and 0.45 mm/y in women and men, respectively [9]. However, in the case presented here, a 0.9 cm growth in aortic diameter was observed within 6 months, which is much faster than that reported previously. This is while our patient did not have the risk factors associated with fast growth and/or dissection, such as hypertension and smoking [10]. This finding confirms the complex etiology of TAA [11]. Besides, our case emphasizes the association between NCLV and fast growing TAA.

Genetic factors have been suggested as one of the possible factors affecting the growth rate of TAA and rupture [12]. The strong association of genes with TAA suggested the heritable pathogenesis, resulting from an innate defect, associated with other heritable cardiac malformations, such as patent ductus arteriosus, ventricular septal defects, aortic and mitral valve abnormalities [13], which has been associated with aggressive growth [11]. The co-occurrence of LVNC and TAA in our case may also suggest the role of genetics. Nevertheless, we could not find evidence on the association of the mutations associated with LVNC [like *HCN4* p.G482R and *MYH11* (MIM 160,745)] [14–16] in the present case. Therefore, we cannot conclude definitely whether the developed TAA in our patient was inherited or acquired, although its association with other cardiac conditions and lack of atherosclerotic risk factors suggest the heritable type. Considering LVNC, the mutations found in the present case, *TTN*, *DSP*, and *DSC2* have been considered as important mutations in LVNC, associated with poorer prognosis [17, 18]. In addition, in the case presented here, the late presentation of LVNC without previous clinical symptoms suggests the gradual development of acquired LVNC; however, we did not have the previous cardiac imaging results to know for sure.

Our case presented here, was another rare phenomenon, as there are only a few reports of aortic aneurysm or dissection associated with LVNC in the literature [19, 20], which were not fast-growing, and PFO, a benign anatomical variant, observed in about 25% of the general population [21], has not been mentioned as a common cardiac malformation associated with LVNC [22]. However, the association of LVNC with PFO results in a higher risk of ischemic stroke [23] and thus adds to the clinical significance of these conditions [24, 25].

This report shows the necessity to pay greater attention to the fast-growing TAA, especially in the presence of other cardiac diseases, which may conceal the diagnosis of TAA by clinical symptoms. Although imaging modalities can help accurate diagnosis, in cases with fast-growing TAA, we should not wait for imaging signs of dissection and/or rupture. Moreover, it should be kept in mind that negative gross findings cannot rule out the possibility of subclinical microscopic dissection in the context of a fast-growing TAA. Therefore, early cardiac surgery (aortic replacement) is highly recommended for saving the patient’s life in such cases. However, because this is a unique case report, further studies and more cases are required to conclude this final result as well.

## Data Availability

The datasets used and/or analyzed during the current study are available from the corresponding author on reasonable request.

## Abbreviations

TAA: Thoracic aortic aneurysm
TTE: Transthoracic echocardiography
TEE: Transesophageal echocardiography
CTA: Computer tomographic angiography
CMR: Cardiac magnetic resonance
LV: Left ventricular
LVNC: Left ventricular non-compaction
PFO: Patent foramen ovale
LVEF: Left ventricular ejection fraction
WES: Whole-exome sequencing
CCTA: Computed tomographic coronary angiography
ICD: Implantable cardioverter defibrillator
CPB: Cardiopulmonary bypass

## References

[1] Mathur A, Mohan V, Ameta D, Gaurav B, Haranahalli P (2016). Aortic aneurysm. J Transl Int Med.

[2] Kuzmik GA, Sang AX, Elefteriades JA (2012). Natural history of thoracic aortic aneurysms. J Vasc Surg.

[3] Saliba E, Sia Y, Dore A, El Hamamsy I (2015). The ascending aortic aneurysm: When to intervene?. Int J Cardiol Heart Vasc.

[4] Guo MH, Appoo JJ, Saczkowski R, Smith HN, Ouzounian M, Gregory AJ (2018). Association of mortality and acute aortic events with ascending aortic aneurysm: a systematic review and meta-analysis. JAMA Netw Open.

[5] Elefteriades JA, Sang A, Kuzmik G, Hornick M (2015). Guilt by association: paradigm for detecting a silent killer (thoracic aortic aneurysm). Open Heart.

[6] Ueda T, Chin A, Petrovitch I, Fleischmann D (2012). A pictorial review of acute aortic syndrome: discriminating and overlapping features as revealed by ECG-gated multidetector-row CT angiography. Insights Imaging.

[7] Boodhwani M, Andelfinger G, Leipsic J, Lindsay T, McMurtry MS, Therrien J (2014). Canadian Cardiovascular Society position statement on the management of thoracic aortic disease. Can J Cardiol.

[8] Cheung K, Boodhwani M, Chan KL, Beauchesne L, Dick A, Coutinho T (2017). Thoracic aortic aneurysm growth: role of sex and aneurysm etiology. J Am Heart Assoc.

[9] Boczar KE, Cheung K, Boodhwani M, Beauchesne L, Dennie C, Nagpal S (2019). Sex differences in thoracic aortic aneurysm growth: role of aortic stiffness. Hypertension.

[10] Landenhed M, Engström G, Gottsäter A, Caulfield MP, Hedblad B, Newton-Cheh C (2015). Risk profiles for aortic dissection and ruptured or surgically treated aneurysms: a prospective cohort study. J Am Heart Assoc.

[11] Booher AM, Eagle KA (2011). Diagnosis and management issues in thoracic aortic aneurysm. Am Heart J.

[12] Goldfinger JZ, Halperin JL, Marin ML, Stewart AS, Eagle KA, Fuster V (2014). Thoracic aortic aneurysm and dissection. J Am Coll Cardiol.

[13] Chen MH, Choudhury S, Hirata M, Khalsa S, Chang B, Walsh CA (2018). Thoracic aortic aneurysm in patients with loss of function Filamin A mutations: clinical characterization, genetics, and recommendations. Am J Med Genet A.

[14] Vermeer AM, Lodder EM, Thomas D, Duijkers FA, Marcelis C, van Gorselen EO (2016). Dilation of the aorta ascendens forms part of the clinical spectrum of HCN4 mutations. J Am Coll Cardiol.

[15] Hanania HL, Regalado ES, Guo D-C, Xu L, Demo E, Sallee D (2019). Do HCN4 variants predispose to thoracic aortic aneurysms and dissections?. Circ Genom Precis Med.

[16] Millat G, Janin A, de Tauriac O, Roux A, Dauphin C (2015). HCN4 mutation as a molecular explanation on patients with bradycardia and non-compaction cardiomyopathy. Eur J Med Genet.

[17] Lorca R, Martín M, Pascual I, Astudillo A, Díaz Molina B, Cigarrán H (2020). Characterization of left ventricular non-compaction cardiomyopathy. J Clin Med.

[18] Corrado D, Basso C, Judge DP (2017). Arrhythmogenic cardiomyopathy. Circ Res.

[19] D'Angeli I, Sürder D, Pedrazzini GB, Moccetti T (2010). Type B aortic dissection in a patient with unknown left ventricular non-compaction cardiomyopathy: cardiovascular magnetic resonance diagnosis. Int J Cardiol.

[20] Esmaeilzadeh M, Moshkani-Farahani M, Abtahi F, Momtahen M (2009). Non-compaction of the ventricular myocardium associated with aortic aneurysm and severe aortic insufficiency: initial description in two cases. Iran Heart J.

[21] Horlick E, Kavinsky CJ, Amin Z, Boudoulas KD, Carroll JD, Hijazi ZM (2019). SCAI expert consensus statement on operator and institutional requirements for PFO closure for secondary prevention of paradoxical embolic stroke: The American Academy of Neurology affirms the value of this statement as an educational tool for neurologists. Catheter Cardiovasc Interv.

[22] Kayvanpour E, Sedaghat-Hamedani F, Gi W-T, Tugrul OF, Amr A, Haas J (2019). Clinical and genetic insights into non-compaction: a meta-analysis and systematic review on 7598 individuals. Clin Res Cardiol.

[23] Pöyhönen P, Kuusisto J, Järvinen V, Pirinen J, Räty H, Lehmonen L (2020). Left ventricular non-compaction as a potential source for cryptogenic ischemic stroke in the young: a case-control study. PLoS ONE.

[24] Wengrofsky P, Armenia C, Oleszak F, Kupferstein E, Rednam C, Mitre CA (2019). Left ventricular trabeculation and noncompaction cardiomyopathy: a review. EC Clin Exp Anat.

[25] Bennett CE, Freudenberger R (2016). The current approach to diagnosis and management of left ventricular noncompaction cardiomyopathy: review of the literature. Cardiol Res Pract.

